# Education in Anthropology

**Published:** 1895-12-21

**Authors:** 


### √ The Study of Man.

### EDUCATION IN ANTHROPOLOGY.

In Egypt, in Greece, in India, we have now the spectacle of races who were found more than adequately equipped with a culture indigenous and appropriate, but who are in danger of being ruined intellectually and morally by the very facility with which they have accepted a Western "education," without acquiring the Western forms of self-respect and self-control. Indeed, it frequently happens that the knowledge of reading or writing stamps a candidate as unfit for employment in a position of trust or authority, and that the prevailing suspicion of literate natives makes their accomplishments a positive drawback to them.

But the most obvious grievance, and at the same time one of the most difficult to remedy, occurs in the attempt to adjust the protectorates exercised admittedly in their own interest by European Powers to the political and legal institutions of "lower races," which are not necessarily unworkable because they are indigenous. These protectorates almost invariably follow series of individual incursions of adventurers, traders, and settlers, and exist for the purpose of legitimising and regulating what, from the point of view of the native, is too often mere spoliation. One would have thought that it was obviously to the interest of the invaders to ascertain the native view of the principle of sovereignty, taxation, retributive justice, property in land, and the like, so that the rules which are to be framed may conflict as little as possible with the existing tradition, and that the native may at least be dispossessed in a way which comes within his view of the rights of conquest or of treaty.

The remedy for all this is not wholly obvious. Much might be done by demanding of candidates for posts which involve contact with native races some elementary knowledge—outside the ordinary run of classical education—of the elements of comparative anthropology in general, especially of comparative sociology and theology; much, too, by giving greater weight to practical experience as against merely theoretical learning.

But so far are we even from the beginnings of such a system, that though of all countries in the world we have by far the finest opportunities of accumulating the materials for the practical study of these questions, we are among the worst equipped with any sort of educational plant for such a purpose. Some hope was once entertained that the Imperial Institute might among many other functions supply something of this need; but in the eight years since its foundation it has done less for this than for any other subject; though clearly the right comprehension and demonstration of this must underlie any scheme for the exploitation of the less developed parts of our empire.

What means we have may be only too briefly reviewed. The British Museum possesses one of the largest and most valuable anthropological collections in the world, and a small but highly qualified staff of specialists. But the staff is so small, and the collection so large, that the mere labour of registering and arranging arrears and new acquisitions absorbs a very large part of their time; and the space allotted to this section of the museum, though itself considerable, is totally inadequate to the proper arrangement and exhibition even of the existing collections—much more to the utilisation of them as an educational rather than a reference collection. Any attempt, moreover, to *invite* contributions, on any considerable scale, so as to make the British Museum really representative of the British Empire in this respect, would result, with the existing accommodation, in immediate overcrowding and chaos.

"South Kensington," needless to say, professes the study of man, as it professes the study of everything else, but with ideals and limitations of its own which are very far from allowing it to take the lead in the most necessary directions; and the almost total want of effective communication between the storehouses of the British Museum, the teaching organisation of South Kensington, and the administrative authority at the Education Office—an anomaly which is not peculiar to this section—necessarily paralyses the over-elaborate mechanism which is supposed to provide national tuition in any such subject.

In the Universities matters are a little better, simply because the machinery is of simpler and more modern pattern. Oxford has a magnificent collection—the Pitt-Rivers Museum—designed by the founder on a comparative system which is of very high educational value, well manned, and very fairly fore-armed against the influx of new material. The Cambridge collections have depended much more upon acquisitions through specialists, and consequently are of a very fragmentary character. Moreover, they are miserably stacked in sheds, which one can only hope are meant to be temporary.

In Oxford and Cambridge, as in London, there is no lack of advanced tuition of a very high order, but students are few, and mostly not of the kind which turns its learning to practical account; partly because in Oxford, at all events, the University has refused to recognise the subject as an avenue, parallel with chemistry or botany, to the B.A. degree; and partly because the present Civil Service rules fail to allow anthropological study as a qualification for the careers where it bears its best fruit.

Very much more might, however, be done in both Universities—and the same applies to a number of other educational bodies with more excuse for omitting it—in making anthropological teaching accessible, as at least a bypath, to those whose circumstances determine them to devote themselves to kindred but more specialised subjects, such as history or "Literæ Humaniores," of which the latter are to a large and increasing degree taking their place as the very specially Greek and Roman subsections of general anthropology; and finding on this side their most promising and extensive development as a practical educational course, in particular as a discipline and exercise in the methods which are applicable to the study of man at large. The Home and Indian Civil Services are now more largely than ever before recruited from students of these subjects, and it is probably on these lines that, in the Universities at all events, strictly anthropological training will eventually be introduced into the University course, as an indispensable element in all really practical training, not as a rival "school" to either natural science, modern history, or "greats."

